# Core-Sheath Structured Yarn for Biomechanical Sensing in Health Monitoring

**DOI:** 10.3390/biomimetics10050304

**Published:** 2025-05-09

**Authors:** Wenjing Fan, Cheng Li, Bingping Yu, Te Liang, Junrui Li, Dapeng Wei, Keyu Meng

**Affiliations:** 1 School of Instrumentation and Optoelectronic Engineering, Beihang University, Beijing 100191, China; fanwenjing@cjlu.edu.cn (W.F.); liangte@buaa.edu.cn (T.L.); lijunrui@buaa.edu.cn (J.L.); 2 College of Metrology Measurement and Instrument, China Jiliang University, Hangzhou 310018, China; s24020804075@cjlu.edu.cn; 3 Chongqing Key Laboratory of Generic Technology and System of Service Robots, Chongqing Institute of Green and Intelligent Technology, Chinese Academy of Sciences, Chongqing 400714, China; 4 School of Electronic and Information Engineering, Changchun University, Changchun 130022, China

**Keywords:** functional yarns, strain sensor, MXene, wearable electronics, health monitoring

## Abstract

The rapidly evolving field of functional yarns has garnered substantial research attention due to their exceptional potential in enabling next-generation electronic textiles for wearable health monitoring, human–machine interfaces, and soft robotics. Despite notable advancements, the development of yarn-based strain sensors that simultaneously achieve high flexibility, stretchability, superior comfort, extended operational stability, and exceptional electrical performance remains a critical challenge, hindered by material limitations and structural design constraints. Here, we present a bioinspired, hierarchically structured core-sheath yarn sensor (CSSYS) engineered through an efficient dip-coating process, which synergistically integrates the two-dimensional conductive MXene nanosheets and one-dimensional silver nanowires (AgNWs). Furthermore, the sensor is encapsulated using a yarn-based protective layer, which not only preserves its inherent flexibility and wearability but also effectively mitigates oxidative degradation of the sensitive materials, thereby significantly enhancing long-term durability. Drawing inspiration from the natural architecture of plant stems—where the inner core provides structural integrity while a flexible outer sheath ensures adaptive protection—the CSSYS exhibits outstanding mechanical and electrical performance, including an ultralow strain detection limit (0.05%), an ultrahigh gauge factor (up to 744.45), rapid response kinetics (80 ms), a broad sensing range (0–230% strain), and exceptional cyclic stability (>20,000 cycles). These remarkable characteristics enable the CSSYS to precisely capture a broad spectrum of physiological signals, ranging from subtle arterial pulsations and respiratory rhythms to large-scale joint movements, demonstrating its immense potential for next-generation wearable health monitoring systems.

## 1. Introduction

The rapid advancement of wearable flexible electronics in recent years has significantly transformed multiple application domains, including health monitoring, soft robotics, and human–machine interaction systems. This transformative potential stems from their unique ability to seamlessly integrate into daily life while enabling continuous, real-time acquisition of physiological data [1,2,3]. Beyond conventional rigid electronics, emerging flexible sensor materials—such as conductive polymers (e.g., PEDOT:PSS), liquid metal, and bio-compatible hydrogels—have expanded the horizons of wearable healthcare systems, enabling real-time monitoring of vital signs, muscle activity, and even biochemical markers [4,5,6]. Within this technological landscape, textile electronics have emerged as a particularly promising branch of flexible devices, garnering substantial research interest due to their inherent advantages, including superior wearer comfort, excellent breathability, and exceptional compatibility with conventional clothing systems. These characteristics collectively position textile electronics as an ideal platform for next-generation wearable applications [7,8,9,10]. Inspired by the hierarchical and adaptive structures found in biological systems, such as the vascular bundles in plant stems that combine mechanical support with flexibility, researchers have sought to mimic these natural designs in sensor development. The fundamental building blocks of textile electronics are the functional yarns that are woven or knitted into fabric structures [11,12,13]. In other words, the characteristics of these yarns are crucial as they directly influence the properties of the fabrics and, consequently, the functionality of the fabricated electronic devices. Given their pivotal role, yarn-based textile sensors are increasingly viewed as viable candidates for the next generation of smart wearable devices.

Among the various types of yarn-based sensors, which operate based on principles such as piezoelectric [14,15], capacitive [16,17], resistive [12,18], and triboelectric effects [19,20], resistive strain sensors have attracted attention due to their high sensitivity, simple construction, and low manufacturing cost. These sensors exhibit a change in electrical resistance in response to mechanical deformation, rendering them particularly suitable for monitoring human physiological signals and movements.

In the pursuit of designing fabric strain sensors with superior overall performance, extensive research has been dedicated to exploring sensitive materials, yarn substrates, and sensor architectures. Inspired by the hierarchical and flexible structure of plant stems, which provide both mechanical strength and flexibility, researchers have sought to mimic these natural designs in sensor development. Notably, the selection of sensitive materials represents a pivotal factor in determining sensor performance. While MXenes are extensively studied in recent research due to their exceptional conductivity and hydrophilicity, alternative nanomaterials such as graphene, carbon nanotubes (CNTs), and conductive polymers are also widely investigated, each offering unique advantages in terms of flexibility, biocompatibility, or scalability. MXene, a new class of two-dimensional materials expressed by M*_n_*_+1_X*_n_*T*_x_*—where M denotes an early transition metal, X represents carbon or nitrogen, T indicates hydrophilic functional groups, and *n* ranges from 1 to 4—has emerged as a hotspot of the research [21,22,23,24]. Ti_3_C_2_, as a representative MXene, offers unique properties like layered structure, ultrahigh electrical conductivity, and high hydrophilicity, making it an excellent candidate for use in sensitive material. Additionally, other conductive nanomaterials, including silver nanowires (AgNWs) and graphene oxide (GO), have been incorporated into textile sensors to enhance performance, demonstrating the versatility of material choices in wearable electronics.

Moreover, the choice of textile substrate and the structure design of the sensor are decisive factors that influence the detection range and the potential application scenarios [25,26,27,28]. The core-sheath architecture observed in plant stems, featuring a rigid central core surrounded by a flexible outer layer, has inspired the development of yarn-based sensors that successfully balance mechanical strength with elastic deformation capabilities. For strain sensors targeting large deformation ranges, elastomeric polymer substrates such as polydimethylsiloxane (PDMS), rubber, and thermoplastic polyurethane (TPU) are particularly suitable due to their inherently low elastic modulus [29,30]. Among these options, rubber has emerged as a preferred substrate material owing to its cost-effectiveness, environmental stability, high elasticity, and impact resistance. In recent years, textile sensors with different structures made from MXene-based functional yarns and different elastic substrates have been widely studied. For instance, Chao et al. developed a flexible wearable tile-like stacked hierarchical MXene/PANIF nanocomposites-based strain sensor for human motion monitoring by coating MXene sheets and PANIF on an elastic rubber substrate [31]. Cai et al. constructed a Ti_3_C_2_T_x_ MXene/Carbon nanotube composite-based strain sensor for phonations, walking, running, and jumping through a layer-by-layer spray coating technique on a latex substrate [32]. Despite the progress and the enhanced comfort provided by MXene-based textile strain sensors in medical monitoring, challenges persist in developing sensors that integrate high sensitivity, broad detection range, low detection limits, long-term durability, and low production costs. These sensors often excel in specific performance areas but fall short in others, underscoring the necessity for a more balanced approach in sensor design to fully capitalize on their potential across a broad array of applications [33,34,35].

In this work, core-sheath structured yarn is fabricated to address the challenges in developing highly flexible and stretchable yarn-shaped sensors with outstanding electrical properties. Drawing inspiration from the natural design of plant stems, which feature a rigid core surrounded by a flexible outer layer, the sensor incorporates a rubber core for elasticity and mechanical strength, surrounded by a cotton yarn sheath dip-coated with Ti_3_C_2_-MXene for conductivity. A rubber core provides elasticity and mechanical strength, surrounded by a cotton yarn sheath dip-coated with Ti_3_C_2_-MXene for conductivity. This is further enhanced by spray-coating silver nanowires (AgNWs) onto the MXene-coated cotton yarn, creating a highly conductive network. An additional cotton yarn layer encapsulates the structure, maintaining elasticity and protecting the conductive network. Flexible silver conductive yarns are stitched to both ends of the sensor for signal transmission. The yarn can elongate by 230%, inducing significant resistance changes. The CSSYS features a low strain detection limit of 0.05%, high sensitivity with a gauge factor of 744.45, a fast response time of 80 ms, a broad detection range, and superior stability over 20,000 cycles, making it ideal for monitoring physiological signals.

## 2. Materials and Methods

### 2.1. Materials

Ti_3_AlC_2_ MAX powder (300 mesh) was purchased from Mingshan Technologies Co., Ltd. (Changchun, China). Hydrochloric acid (HCl) and lithium fluoride (LiF) were purchased from Alfa Aesar Chemical Co., Ltd. (Shanghai, China). AgNWs were purchased from Aladdin Co., Ltd. (Shanghai, China). Rubber and cotton yarns were purchased from Bayashipin Textile Co., Ltd. (Guangzhou, China). The conductive silver yarn was purchased from Shenzhen Xinwei Electronic Materials Co., Ltd. (Shenzhen, China).

### 2.2. Synthesis of Monolayered Ti_3_C_2_T_x_ MXene

Ti_3_C_2_T_x_ MXene nanosheets were prepared by etching commercial Ti_3_AlC_2_-MAX using a mixture of HCl and LiF. First, 1 g of LiF was dissolved in 20 mL of HCl in a Teflon vessel at room temperature. The solution was stirred at 35 °C for 20 min. Subsequently, 1 g of Ti_3_AlC_2_ was slowly added to the mixture and stirred continuously at 35 °C for 36 h. The suspension was washed repeatedly with deionized (DI) water until the pH reached 6–7, yielding multilayered Ti_3_C_2_T_x_ MXene. Finally, the mixture was centrifuged at 3500 rpm for 1 h, resulting in the separation and collection of monolayered Ti_3_C_2_T_x_ MXene nanosheets.

### 2.3. Fabrication of the MXene-Based Textile Pressure Sensor

**Figure 1:**
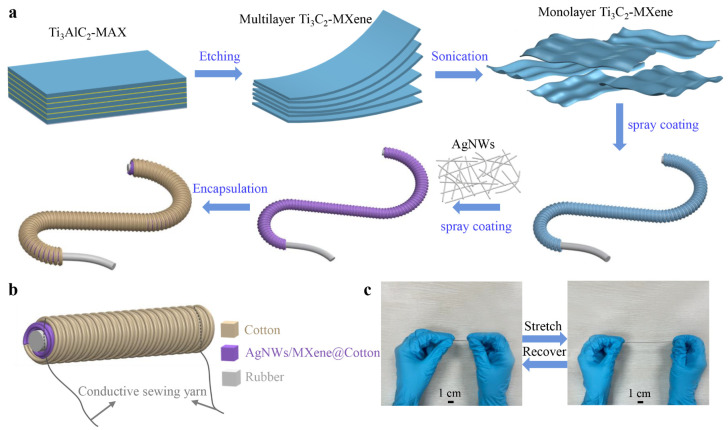
Fabrication and photograph of the core-sheath structured yarn strain sensor. (**a**) Fabrication process of the yarn sensor. (**b**) Schematic diagram of the yarn sensor. (**c**) Photograph of the change in elongation of the yarn sensor.

The fabrication methodology, as schematically illustrated in Figure 1a, commences with the chemical etching of Ti_3_AlC_2_-MAX phase precursor to yield multilayer Ti_3_C_2_-MXene, which subsequently undergoes delamination through controlled sonication to produce high-quality monolayer Ti_3_C_2_-MXene nanosheets with well-preserved structural integrity. Drawing inspiration from the biomechanical synergy observed in plant stems, where a rigid core is enveloped by a pliable sheath to achieve both strength and adaptability, the yarn architecture features an elastic rubber core that provides essential mechanical robustness and reversible elasticity. The yarn architecture features an elastic rubber core that provides both essential mechanical robustness and reversible elasticity. A primary cotton sheath is helically wound around the core, which undergoes dip-coating with monolayer Ti_3_C_2_-MXene to establish a uniform and continuous foundational conductive layer. To further optimize electrical conductivity and enhance charge transport pathways, silver nanowires (AgNWs) are uniformly deposited through a precisely controlled spray-coating process, forming an interconnected and synergistic conductive network that complements the MXene coating. Finally, the structure is encapsulated with an outer cotton yarn layer that serves dual purposes: (1) providing robust mechanical protection for the underlying conductive layers against environmental and operational stresses, while (2) significantly enhancing the yarn’s overall structural integrity without compromising its inherent elastic properties. This bioinspired hierarchical design, reminiscent of the natural integration of strength and flexibility in plant tissues, ensures exceptional stability of the conductive network even under substantial and repeated mechanical deformation. The carefully engineered hierarchical design ensures exceptional stability of the conductive network even under substantial and repeated mechanical deformation.

As shown in Figure 1b, flexible silver conductive yarns are strategically stitched at both termini of the sensor to establish reliable electrical connections for signal transmission to measurement equipment. A critical design feature involves the deliberate penetration of these silver electrodes through both the MXene and AgNWs conductive layers to ensure optimal electrical contact and minimal interface resistance. The resultant core-sheath structured yarn exhibits exceptional elongation capability, achieving strains of up to 230%, which induces a pronounced and highly measurable change in electrical resistance—this strain-dependent resistive response constituting the fundamental sensing mechanism. Notably, this resistive response maintains excellent linearity across the entire operational range, ensuring consistent and reliable performance under varying deformation conditions.

### 2.4. Working Mechanism and Morphology of the CSSYS

Understanding the working mechanism of strain sensors is fundamental to optimizing their performance for health monitoring applications. Resistive strain sensors typically rely on conductive network deformation to generate measurable resistance changes under mechanical strain. However, achieving both high sensitivity and wide detection range remains challenging due to trade-offs between material conductivity and structural design. The CSSYS addresses this challenge through its bioinspired core-sheath architecture, which mimics the hierarchical organization of natural systems to enable efficient strain transduction while maintaining structural integrity.

**Figure 2:**
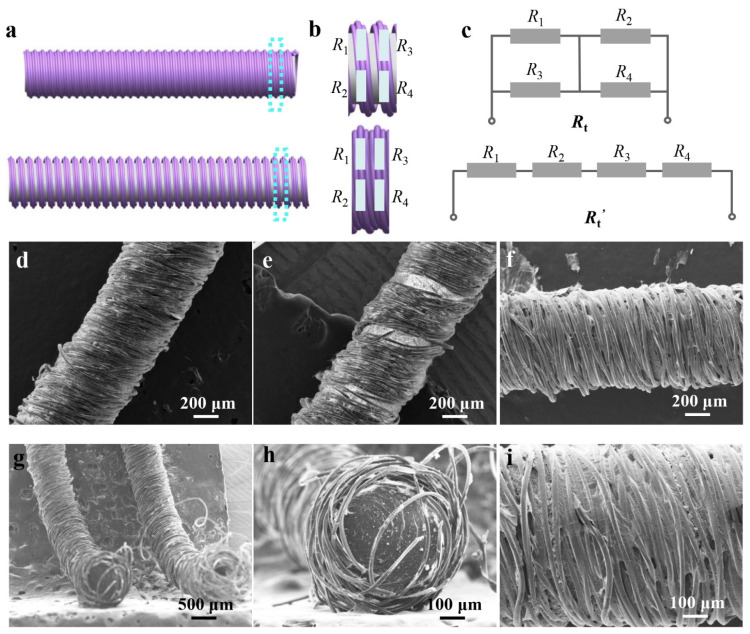
The working mechanism and morphology of the core-sheath structured yarn sensor. (**a**) The original and extended state of the core-sheath structured yarn. (**b**) The equivalent resistance of the yarn. (**c**) The equivalent resistance network of the yarn in its initial and stretched state, respectively. (**d**) The morphology of the original yarn in its initial state. (**e**) The morphology of the original yarn in its extended state. (**f**) The SEM image of the AgNWs/MXene-coated yarns. (**g**) The cross-sectional SEM image of the CSSYS. (**h**) The enlarged view of the cross-section of the CSSYS. (**i**) The enlarged view of the AgNWs/MXene-coated yarns.

To elucidate the working mechanism of the CSSYS, a geometric weave model has been established, as shown in Figure 2a. In the initial state, the fibers form a tightly interwoven network where MXene nanosheets and AgNWs maintain intimate contact through overlapping conductive regions and interparticle electron transport pathways. This dense packing creates multiple parallel conduction channels with low interfacial resistance. Under tensile strain, the structural rearrangement increases inter-fiber spacing, progressively breaking MXene-MXene contacts. The resulting separation reduces effective conduction cross-sections, transforms parallel pathways into serial connections, and introduces additional tunneling barriers, collectively increasing the network resistance proportionally with strain magnitude.

To demonstrate the changes in the resistance of the CSSYS, it is assumed that the resistance of a representative yarn segment is modeled using four discrete resistances (*R*_1_, *R*_2_, *R*_3_, and *R*_4_) (Figure 2b). Based on the working principle of the CSSYS, the equivalent resistance circuit of the sensor is constructed, as illustrated in Figure 2c. Initially, the total resistance of the CSSYS is determined by the series and parallel relationship of these four resistances, and can be represented as

(1) $$R_{t}=\frac{R_{1}R_{3}}{R_{1}{+R}_{3}}+\frac{R_{2}R_{4}}{R_{2}{+R}_{4}}$$

When the CSSYS is stretched, *R*_1_ and *R*_3_ separate simultaneously with *R*_2_ and *R*_4_, the original series and parallel relationship transform into a simple series relationship. Consequently, the resistance of the CSSYS can be expressed by

*R*_t_*’* = *R*_1_ + *R*_2_ + *R*_3_ + *R*_4_ (2)

Based on the results of the mathematical comparison, when the four equivalent resistances are equal and greater than 1 Ω, *R*_t_*’* is always greater than *R*_t_. Given that the intrinsic resistance of the sensitive materials is significantly higher than 1 Ω, the total resistance of the CSSYS after stretching is consistently greater than the resistance in its initial state, validating the strain-responsive behavior.

**Figure 3:**
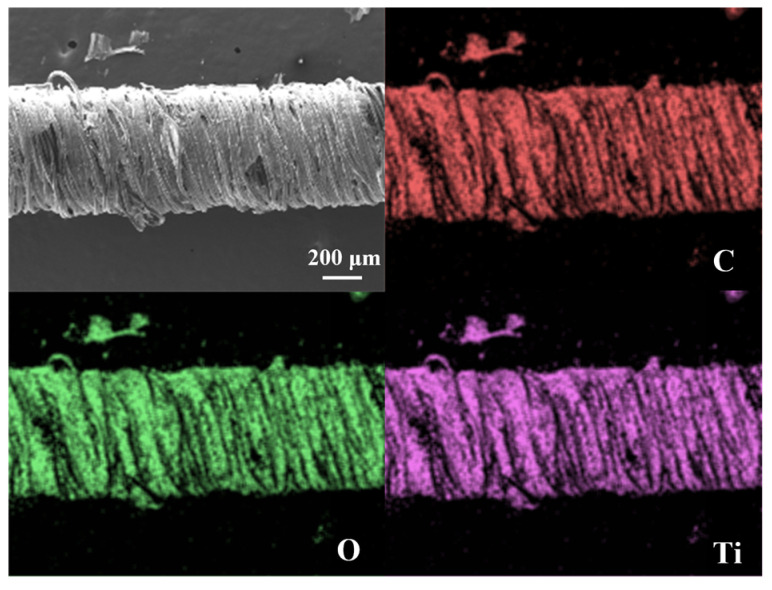
Energy-dispersive spectroscopy mapping of the MXene-coated yarn.

Figure 2d,e depict the microstructural evolution of the CSSYS in its pre-stretch and post-stretch states, respectively. The post-stretch images clearly demonstrate fiber separation, confirming the disruption of conductive pathways and supporting the resistance change mechanism. The surface morphology of the sensor, coated with MXene and AgNWs, is depicted in Figure 2f, with an enlarged view in Figure 2i highlighting the conductive bridges formed by AgNWs on the MXene matrix. The scanning electron microscope (SEM) image in Figure 2g, along with the cross-sectional SEM image of the sensor in Figure 2h, reveals that the inner rubber of the sensor is tightly enveloped by the outer cotton yarns. Furthermore, the energy dispersive spectroscopy (EDS) map (Figure 3) confirms the uniform distribution of key elements (C, Ti, O) from MXene across the surface of the MXene-coated yarn, ensuring consistent conductive properties.

This mechanism is further validated through equivalent circuit modeling and microscopic characterization. The combination of experimental and theoretical analysis provides comprehensive insights into the sensor’s strain-responsive behavior, which is crucial for its application in detecting both subtle physiological signals and large joint movements.

## 3. Results

### 3.1. Characterization of the CSSYS

The performance characterization of wearable strain sensors is crucial for determining their suitability in health monitoring applications. Key parameters including sensitivity, detection range, response time, and durability directly impact a sensor’s ability to accurately capture physiological signals ranging from subtle pulses (<1% strain) to large joint movements (>50% strain). Recent advances in flexible electronics have pushed the boundaries of these performance metrics, yet achieving an optimal balance remains challenging due to inherent trade-offs between material properties and structural design. The CSSYS’s unique core-sheath architecture, combining MXene nanosheets and AgNWs in a biomimetic configuration, represents a significant step toward overcoming these limitations by synergistically enhancing multiple performance characteristics.

In order to thoroughly assess the performance of the CSSYS across multiple dimensions, an efficient measurement system was established, consisting of a horizontal tensile testing machine (ZQ-990L, Zhiqu Technology, Guangzhou, China) for precise strain application, a high-precision multifunctional digital sampling multimeter (DMM7510, Tektronix, Shenzhen, China) for accurate resistance measurement, and a dedicated testing machine controller/data acquisition computer for synchronized data collection and analysis.

The comprehensive evaluation approach adopted here addresses both fundamental sensor characteristics and practical operational requirements. By systematically examining static and dynamic responses under various conditions, we demonstrate how the CSSYS’s structural design translates into superior sensing capabilities that meet the rigorous demands of continuous health monitoring.

As a fundamental performance metric for strain sensors, sensitivity was quantitatively evaluated through the gauge factor (GF). The GF of a sensor is defined as [36]

**Figure 4:**
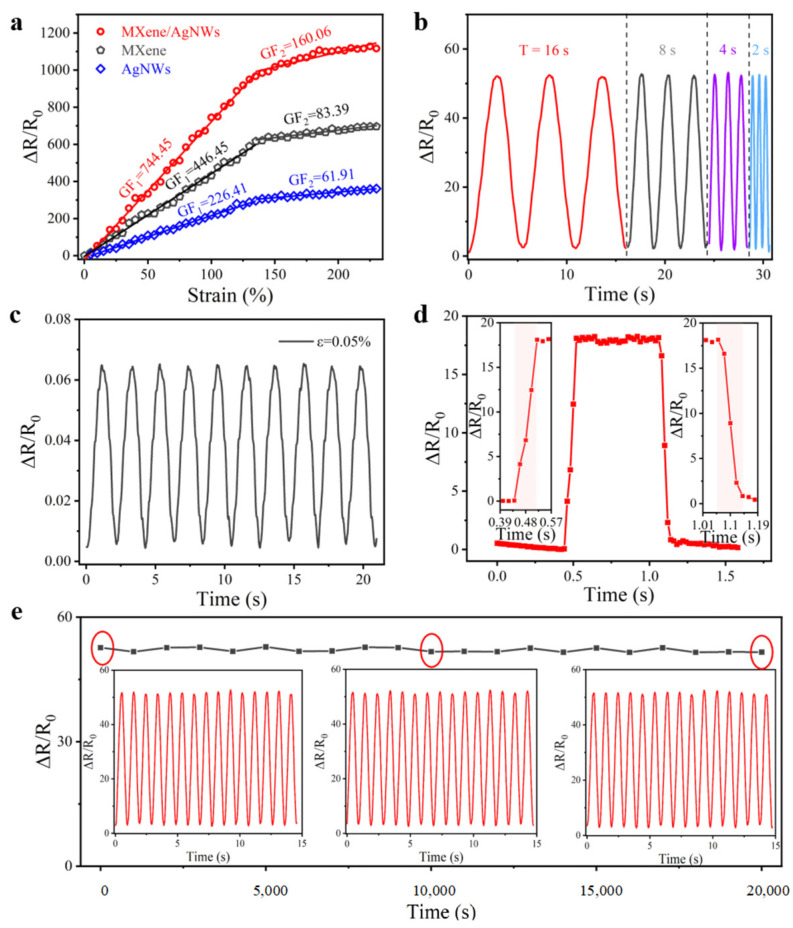
Sensing performance of the CSSYS. (**a**) Typical response−strain curves of the CSSYS. (**b**) Response variability of the CSSYS across various frequencies (*f* = 1/16 Hz, 1/8 Hz, 1/4 Hz, 1/2 Hz). (**c**) ∆R/R_0_ change of the CSSYS under a repeated slight strain of 0.05%. (**d**) Response/recovery time of the CSSYS. (**e**) 20,000 tensile cycles of the sensor under a strain of 10%. The insets are the details of the waveforms at different moments.

GF = (*R* − *R*_0_)/*R*_0_*ɛ* = ∆*R*/*R*_0_*ɛ* (3)

where *R*_0_ is the initial resistance without any load, *R* represents the resistance observed during testing, Δ*R* indicates the relative change in resistance when the sensor is stretched, and ɛ is the strain applied to the sensor. Figure 4a illustrates a Δ*R*/*R*_0_-strain curve with two distinct intervals, showing a strain range of 0–135% with a GF of 744.45 and 135–230% with a GF of 160.06, both of which are greater than the values of sensors prepared with only one sensitive material (MXene or AgNWs). The observed performance enhancement can be attributed to the unique structural evolution during stretching: as strain increases, the distance between conductive elements on the fiber substrates progressively expands, with complete separation of all circularly arranged fibers occurring at the 135% strain.

Moreover, the reliability and stability of the sensor under varying operating frequencies were assessed. As illustrated in Figure 4b, the CSSYS maintains consistent resistance response characteristics when subjected to different tensile periods (T = 32, 16, 8, 4 s) at a constant 10% strain. The results demonstrate no significant impact of operating frequency on the resistance change, suggesting that our CSSYS maintains an outstanding dynamic response capability and operational stability under various tensile rates. Figure 4c showcases the sensor’s remarkable sensitivity to minute mechanical stimuli. The CSSYS achieves clear and reproducible signal detection at an ultra-low strain level of 0.5%, demonstrating its potential for precise monitoring of subtle physiological movements and micro-vibrations in wearable applications. In the field of human health monitoring, the response and recovery times of the wearable device are essential. When subjected to an instantaneous 5% strain, the CSSYS exhibits outstanding response dynamics with both response and recovery times measuring 80 ms (Figure 4d). This rapid response performance meets the stringent requirements for real-time physiological signal monitoring. Long-term operational stability was evaluated through rigorous cyclic testing. As illustrated in Figure 4e, the CSSYS maintains exceptional signal consistency through 20,000 continuous stretch–release cycles at 10% maximum strain, with no observable degradation in response characteristics. This outstanding cyclic durability confirms the sensor’s structural integrity and reliable performance for prolonged use in practical applications.

**Figure 5:**
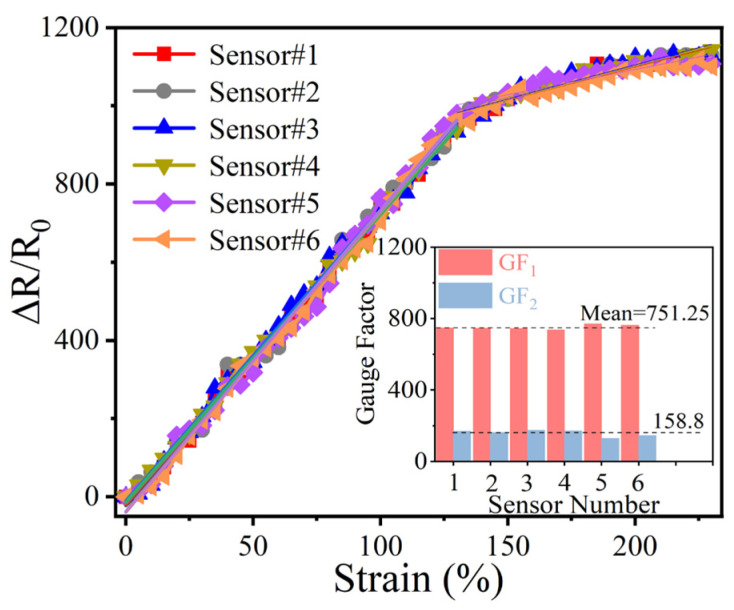
Consistency test of the CSSYS.

To guarantee output consistency in practical applications, a longer segment of the yarn sensor is split into six smaller units, each with the same effective experimental length. The output signal of every sensor is then examined. Under different strains, these sensors displayed a notably uniform electrical output signal, as depicted in Figure 5. The inset of Figure 5 illustrates the GF values of the six sensors, with the GF values of the two intervals remaining within a consistent range. These results not only confirm the excellent batch-to-batch reproducibility but also validate the homogeneous distribution of sensitive materials throughout the substrate, a critical factor for scalable production and practical deployment.

This systematic evaluation demonstrates that the CSSYS achieves an optimal balance of high sensitivity, wide detection range, rapid response/recovery time, and exceptional durability—exhibiting superior performance compared to other reported works, as evidenced in Table 1.

### 3.2. Real-Time Monitoring System for Physiological and Joint Movement Signals

Wearable health monitoring technologies have emerged as a transformative solution to address the growing global burden of chronic diseases and aging populations. According to the World Health Organization, cardiovascular diseases account for over 17.9 million deaths annually, while musculoskeletal conditions affect 1.7 billion people worldwide. Early detection and continuous monitoring of physiological signals—such as pulse waveforms, respiratory patterns, and joint mobility—are critical for preventive healthcare and personalized medicine. Traditional clinical devices (e.g., electrocardiograms, spirometers, and goniometers) are often bulky, expensive, and limited to sporadic measurements in hospital settings. In contrast, textile-based sensors like the CSSYS offer unprecedented advantages by enabling non-invasive, real-time, and long-term monitoring of vital signs during daily activities. This aligns with the paradigm shift toward decentralized healthcare, where patients can actively participate in managing their well-being through wearable technologies.

**Figure 6:**
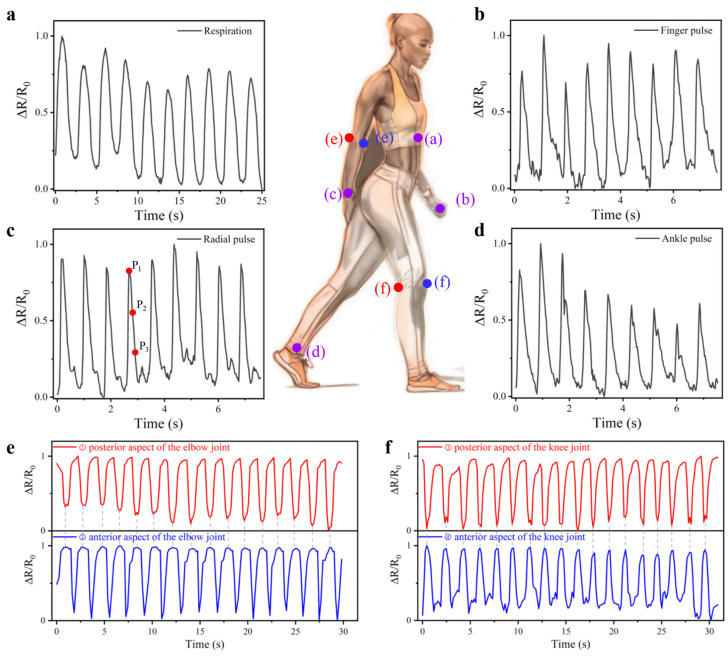
The CSSYS for human physiological and motion signal monitoring. (**a**) Respiration waveform at thoracic cavity. Pulse waveform at the (**b**) finger, (**c**) radial, and (**d**) ankle artery. The activity waveform at (**e**) elbow and (**f**) knee.

The CSSYS demonstrates exceptional multimodal monitoring capabilities (both minute pulse signals and more pronounced joint activities) owing to its superior electrical properties and structural design. When integrated into close-fitting garments, such as undershirts, the CSSYS can effectively capture respiration signals emanating from the thoracic cavity. As shown in Figure 6a, the sensor reliably detects a respiratory rate of 24 beats per minute (BPM), which falls within the normal range (12–20 BPM for adults at rest) with slight elevation potentially indicating mild physical activity. This performance highlights the system’s capability for non-invasive, continuous respiratory monitoring with clinical-grade precision.

Beyond respiration, pulse wave analysis provides a window into cardiovascular health. The shape and timing of arterial pulse waveforms reflect cardiac output, vascular stiffness, and autonomic nervous system activity—key indicators of conditions like hypertension, atherosclerosis, and heart failure. Conventional photoplethysmography (PPG) sensors struggle with motion artifacts and poor signal quality at peripheral sites (e.g., fingers or ankles). The CSSYS overcomes these limitations through its ultra-low detection limit (<0.05% strain) and conformal contact with skin, enabling robust pulse acquisition even during movement.

**Figure 7:**
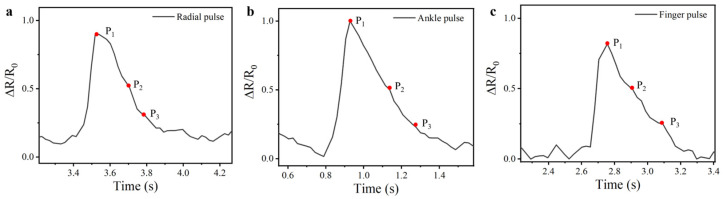
The pulse waveforms at (**a**) radial, (**b**) ankle, and (**c**) finger positions, along with their characteristic points.

Building upon its physiological monitoring capacity, the CSSYS demonstrates its versatility through its integration with various wearable accessories, including finger gloves, wristbands, and socks, enabling real-time pulse signal monitoring at multiple anatomical locations, as shown in Figure 6b–d. With an exceptional sensitivity (detection limit < 0.05% strain), the system reliably acquires pulse signals even at peripheral sites. This is particularly evident in its ability to detect faint finger pulse signals, which are notoriously difficult to capture due to their low amplitude. Despite the anatomical differences, pulse signal contours vary slightly across different body parts. However, the pulse frequency remains largely consistent. Most significantly, as illustrated in Figure 7, the CSSYS precisely identifies key pulse wave characteristic points at different positions: P1 (related to cardiac systolic function), P2 (reflecting arterial stiffness), and P3 (associated with aortic valve closure). The accurate detection of these hemodynamic markers enables comprehensive cardiovascular assessment, including pulse wave velocity calculation and augmentation index determination—critical parameters for early diagnosis of arterial stiffness and other cardiovascular pathologies.

In musculoskeletal rehabilitation, continuous joint motion tracking is essential for assessing recovery progress and preventing re-injury. Existing motion capture systems rely on cameras or inertial sensors, which are impractical for daily use. The CSSYS’s ability to monitor elbow and knee flexion/extension (Figure 6e,f) with high synchrony offers a scalable alternative for tele-rehabilitation and elderly fall prevention. Its dual functionality—combining subtle physiological signal detection with large-motion tracking—positions it as a holistic health monitoring platform.

In addition to monitoring pulse and respiration signals, the CSSYS can also track joint activity by being integrated into supportive garments such as elbow and knee braces, as exhibited in Figure 6e,f. The bilateral placement of sensors on anterior and posterior joint surfaces enables precise quantification of movement dynamics through complementary signal patterns. For instance, an increase in the output signal from the sensor on the anterior side of the knee corresponds with a decrease in the output signal from the posterior side, demonstrating a high synchronization and consistency in monitoring joint activities.

This dual capability of detecting both subtle pulse signals and more substantial joint movements establishes its clinical utility in rehabilitation medicine, where continuous monitoring provides objective metrics for tailoring postoperative recovery protocols and tracking functional improvement. For patients recovering from musculoskeletal injuries or surgeries, continuous and accurate monitoring of joint movements can offer real-time feedback, aiding in tailored rehabilitation protocols and improving recovery outcomes. Furthermore, the exceptional signal fidelity and textile-based design of the CSSYS facilitate its integration into advanced prosthetic interfaces and bio-inspired robotic systems, enabling naturalistic movement intention detection and precise control signal generation. These attributes position the CSSYS as a versatile platform bridging diagnostic monitoring and therapeutic applications in musculoskeletal rehabilitation and assistive technology development.

## 4. Conclusions

In summary, the CSSYS represents an innovative advancement in flexible electronic textiles, featuring a meticulously engineered architecture that begins with the chemical etching of Ti_3_AlC_2_-MAX phase to yield multilayer Ti_3_C_2_-MXene, followed by controlled sonication to obtain monolayer dispersion. The structural design of the CSSYS incorporates an elastic rubber core for mechanical robustness, while a cotton yarn sheath functionalized with monolayer Ti_3_C_2_-MXene through dip-coating establishes the primary conductive pathway, which is further enhanced by spray-coated AgNWs to create a hierarchical conductive network with exceptional electron transport properties. The complete encapsulation with an additional cotton yarn layer ensures both environmental protection and mechanical durability while preserving the intrinsic elasticity of the structure. The fabricated sensor demonstrates outstanding performance characteristics, including an extensive 230% strain capability accompanied by significant resistance variation, ultra-sensitive detection capability down to 0.05% strain, remarkable gauge factor of 744.45, rapid response time of 80 ms, broad detection range, and exceptional cycling stability over 20,000 operational cycles. These superior electrical properties enable the CSSYS to perform multimodal physiological monitoring with high precision, reliably capturing both subtle pulse/respiratory signals and gross joint movement patterns in real time across various anatomical locations, thereby establishing its significant potential as a versatile platform for advanced human health monitoring applications.

## Figures and Tables

**Table 1:** A sensing performance comparison between the CSSYS and other strain sensors.

Sensing Material	Structure	GF (Detect Range)	Response Time (ms)	Minimum Detection Limit	Ref.
MXene/PPy/HEC	Sandwich	118 (0–12%) 357.5 (12–94%)	300	0.5%	[37]
Liquid alloy	Core-shell	0.31 (0–130%) 10.8 (180–300%)	/	/	[38]
Laser-induced Graphene	Knitted	34.8 (0–2.4%) 117.9 (2.4–3%)	192	/	[39]
Graphene	Cracks	317 (<5%)	/	0.05%	[40]
Graphene/Ag NWs	Knitted	9.25 (20–61%)	40	/	[41]
MXene/AgNWs	Core-shell	744.45 (0–135%) 160.06 (135–230%)	80	0.05%	This work

## Data Availability

Data will be made available on request.

## Abbreviations

The following abbreviations are used in this manuscript:

CSSYS	Core-sheath structured yarn sensor
PDMS	Polydimethylsiloxane
TPU	Thermoplastic polyurethane
AgNWs	Silver nanowires
HCL	Hydrochloric acid
LiF	Lithium fluoride
DI	Deionized
SEM	Scanning electron microscope
EDS	Energy dispersive spectroscopy
GF	Gauge factor
BPM	Beats per minute
